# A CMOS Low Pass Filter for SoC Lock-in-Based Measurement Devices

**DOI:** 10.3390/s19235173

**Published:** 2019-11-26

**Authors:** Jorge Pérez-Bailón, Belén Calvo, Nicolás Medrano

**Affiliations:** Group of Electronic Design, Aragon Institute for Engineering Research, I3A, University of Zaragoza, 50009 Zaragoza, Spain; becalvo@unizar.es (B.C.); nmedrano@unizar.es (N.M.)

**Keywords:** low pass filter, very low frequency, lock-in amplifier, impedance spectroscopy, sensor array, low-voltage low-power, on-chip instrumentation

## Abstract

This paper presents a fully integrated *G_m_*–C low pass filter (LPF) based on a current steering *G_m_* reduction-tuning technique, specifically designed to operate as the output stage of a SoC lock-in amplifier. To validate this proposal, a first-order and a second-order single-ended topology were integrated into a 1.8 V to 0.18 µm CMOS (Complementary Metal-Oxide-Semiconductor) process, showing experimentally a tuneable cutoff frequency that spanned five orders of magnitude, from tens of mHz to kHz, with a constant current consumption (below 3 µA/pole), compact size (<0.0140 mm^2^/pole), and a dynamic range better than 70 dB. Compared to state-of-the-art solutions, the proposed approach exhibited very competitive performances while simultaneously fully satisfying the demanding requirements of on-chip portable measurement systems in terms of highly efficient area and power. This is of special relevance, taking into account the current trend towards multichannel instruments to process sensor arrays, as the total area and power consumption will be proportional to the number of channels.

## 1. Introduction

Recent technological advances in the implementation of CMOS-based sensors has raised the interest of designing low-power compact electronic interfaces to be integrated in the same chip as the sensing element, so as to obtain miniaturized system-on-chip (SoC) portable devices with improved reliability and reduced fabrication costs. In this endeavor, there is one crucial front-end basic building block that possess significant challenges in being fully integrated with high performance, compact size, and low-power consumption—low-pass filters (LPFs) with very low cutoff frequencies.

Accordingly, in recent years, there has been significant research efforts towards the development of such LPFs, boosted mainly because of their application in biomedical systems [1,2,3,4], where it is necessary to low pass filter the signal over the frequencies of interest—typically in the 100 mHz to 1 kHz range—to remove noise before digitizing it for further processing. These LPFs are also widely used as DC (Direct Current) magnitude extractors; in this case, they are typically placed in the last stage of the sensor readout chain and require sub-Hz cutoff frequencies, such as in lock-in amplifiers (LIA), an extremely versatile instrument mostly used as a precision AC (Alternating Current) voltage and AC phase meter, or equivalently, as an impedance spectroscope [5,6,7,8,9,10,11,12,13], an application field that is the motivation of this work.

Lock-in amplifiers are based on a technique known as phase sensitive detection (PSD) that can extract the amplitude and phase of a signal even in a noisy environment operating at a known frequency *f*_0_ [14,15,16,17]. In a single-phase LIA, the input signal *V_in_* = a_s_*sin(*ωt*) (sensor response) is typically amplified by a low-noise amplifier (LNA) *A_s_* = A_LNA_*a_s_; then, a mixer or phase-sensitive detector controlled by a reference signal *V_ref_* of the same frequency *f*_0_ and aligned in phase (*θ* = 0) with *V_in_* demodulates the input signal [18]. An output LPF, with a suitable corner frequency, extracts the DC component *V_X_* of the resulting synchronously modulated signal, which is proportional to the input signal amplitude according to

(1) $$V_{X}=\frac{V_{dd}}{2}+\frac{2A_{s}cos\left(\theta \right)}{\pi },$$

assuming that *V_ref_* is a digital signal, *V_dd_* is the system single supply, and *V_dd_*/2 the common mode voltage.

**Figure 1:**
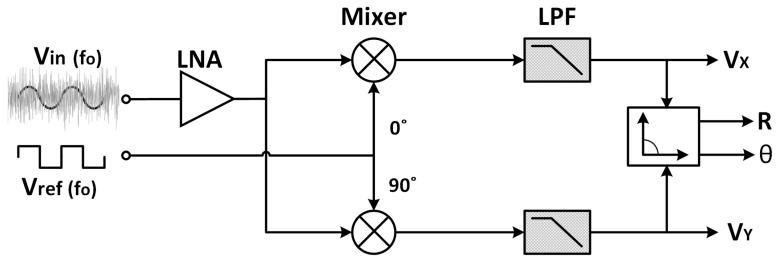
Dual-phase lock-in amplifier.

To recover both amplitude and phase information, a dual-phase LIA with two branches—with two outputs *V_X_* and *V_Y_*, instead of one (Figure 1)—is needed. The input signal is respectively multiplied in both branches by quadrature reference signals *V_ref_* (*f*_o_) and *V_ref_* (*f*_o_, 90°), recovering after the corresponding LPF the DC outputs *V_X_* and *V_Y_*, proportional to the magnitude and phase or, equivalently, the real and imaginary components:

(2) $$V_{x}=\frac{V_{dd}}{2}-\frac{2A_{s}cos\left(\theta \right)}{\pi },$$

(3) $$V_{y}=\frac{V_{dd}}{2}-\frac{2A_{s}sin\left(\theta \right)}{\pi }.$$

Assuming that all the electronics prior to the filter present low noise performance, the accuracy in the recovery largely depends on the LPF cutoff frequency. In this sense, a LIA can be understood as a band-pass filter with central frequency *f*_0_ and a very high quality factor Q = (*f*_0_/*f*_c_), where *f*_c_ is the bandwidth of the output low-pass filter. Hence, the smaller the LPF cutoff frequency, the better the noise rejection and the better the recovery accuracy, but a compromise arises with the related acquisition times.

Different integrated LIAs have been recently proposed for smart instrumentation applications [13,19,20,21] to exploit the advantages that render CMOS compatibility in terms of miniaturization. However, these LIAs maintain the LPF external use of off-chip resistors and capacitors [19,20,21] or, for fully integrated LIA solutions [13], the active filter area is rather large (it is the dominant element of the 3.6 mm^2^ area of the implemented chip) for frequencies ~300 Hz. In particular, a previous author’s proposal [21] achieves very competitive capabilities in terms of area, power, and signal recovery, but the LPF is also kept external. Thus, the purpose of this work was to design an LPF suitable for this LIA architecture to achieve a fully integrated design. Accordingly, the design specifications were 1.8 V to 0.18 µm CMOS monolithic single-ended stage with two configurable cutoff frequencies of 0.5 and 5 Hz to bring flexibility to the system adjusting the speed-accuracy trade-off; input range from *V_dd_*/2 = 0.9 V up to *V_dd_* = 1.8 V, corresponding to the synchronous rectified signal range, assuming a single supply *V_dd_* = 1.8 V and signals over a common-mode *V_cm_* = *V_dd_*/2; low noise, to preserve high dynamic range; compact size (<0.1 mm^2^) and minimum power consumption (<10 µW) with currents of the order of hundreds of nA to be reliably generated on-chip.

There is a vast amount of literature on integrated low pass filters with very low-cutoff frequencies, mainly based on *G_m_*–C approach [22,23,24,25,26,27,28,29,30,31,32,33,34,35,36] and focused on biological signal processing. Therefore, besides not strictly presenting a tuneable frequency over our target sub-Hz to Hz range (5.4 kHz [22], from 2 kHz to 20 kHz [23]), some of them exhibit a power consumption rather high to be suitable to be integrated within multichannel systems ([24] consumes 75.9 µW, [25] from 59.5 µW to 90 µW, and [26] 105.3 µW including a buffer). Among those that are power-efficient, either area is jeopardized, restricting their use within portable devices (an area of 0.336 mm^2^ is reported in [27], 0.2 mm^2^ in [28,29] has an external 10 nF capacitor, [30] has an area of 1 mm^2^, and an area of 0.24 mm^2^ is reported in [31]), or dynamic range is jeopardized (34 dB [22] and 49.9 dB [32]), whereas others achieve such low power thanks to bias currents in the order of pA or a few nA, which are difficult to be reliably generated on-chip and are typically tuned to adjust the *G_m_* and thus adjust the cut-off frequency (from 300 pA to 900 pA [33], from 90 pA to 430 pA [34], in [35] two bias currents are used ranging from 200 pA to 4 nA and from 1 nA to 20 nA respectively, and from 250 pA to 25 nA [36]), existing on the overall power-area-dynamic range trade-off that makes their design a real challenge.

Thus, a novel low pass filter is needed that satisfies all the needed specifications for its operation as a DC extractor in a portable multichannel LIA-based measurement system, enhancing the state-of-the-art power-area-dynamic range trade-off, so as to obtain a topology suitable for its use in the next generation of lock-in based-measurement devices for impedance sensor arrays.

To do so, we notice that in [1] an OpAmp active-RC low pass filter with a current steering technique (CST) that attenuates the current through the integrator in the feedback loop is proposed, reaching an f_c_ down to 0.25 Hz from a nominal target f_c_ of 18 Hz. It is a simpler structure compared to previously reported *G_m_*–C techniques, but at the expense of a reduced input impedance. Thus, in this work, a *G_m_*–C approach was adopted to attain a high impedance input node, which made the coupling between stages straightforward. The core of the V–I converter remained unaltered so that the bias point was not moved from its optimum value, whereas both *G_m_* reduction and tuning were done in the transconductor output current transfer section, exploiting a current steering technique as the most suitable choice for effectively reducing the *G_m_*, preserving a good overall performance trade-off. In this way, all the requirements of a SoC high performance solution can be simultaneously met, bringing about a very competitive solution.

Authors have reported preliminary simulation results of the basic integrator in [37]. This current paper offers a more in depth study and the complete experimental characterization of these structures. The paper is organized as follows: Section 2 describes the proposed *G_m_* topology and the two derived low pass filters. The experimental results are summarized in Section 3, in Section 4 experimental measurements of the LPF applied to a lock-in amplifier are shown, and conclusions are drawn in Section 5.

## 2. Proposed *G_m_*-C LPF

**Figure 2:**
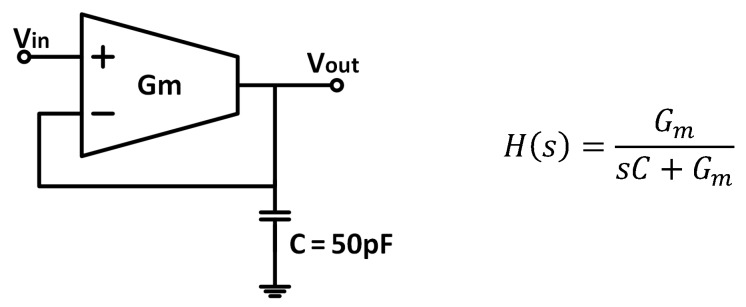
First-order *G_m_*-C low-pass filter (LPF) and its corresponding transfer function.

A single-input single-output LPF was required for our application (Figure 1). Therefore, a differential-input single-output *G_m_*-C architecture in unity gain feedback configuration was adopted. The resulting closed-loop configuration maintained, without a specific *G_m_* linearization technique, good linearity in the passband over all the input range while not degrading the noise [36], optimizing the dynamic range in this way. Note that for this scheme, when the input signal frequency is close to the filter cut-off frequency, there will be an important phase shift among both inputs of the transconductor, which will cause distortion. Therefore, it is not a general-purpose low-pass signal-processing filter, but a DC extractor for synchronously rectified signals operating at higher frequencies. Figure 2 shows the basic order-1 scheme and the corresponding transfer function, with a pole located at *G_m_*/C [38]. The load capacitor value was set to 50 pF, considered the maximum practical on-chip capacitor.

Two structures were implemented: the basic integrator (order-1 filter, O1F) in Figure 2 and a second-order LPF (O2F).

### 2.1. Transconductor Architecture

**Figure 3:**
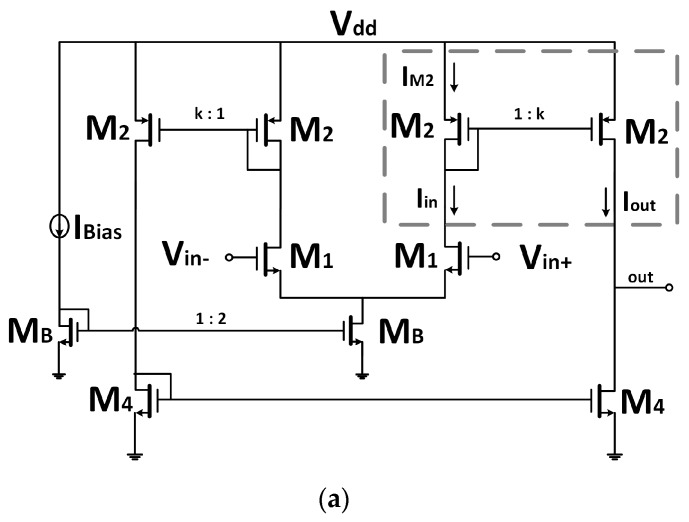

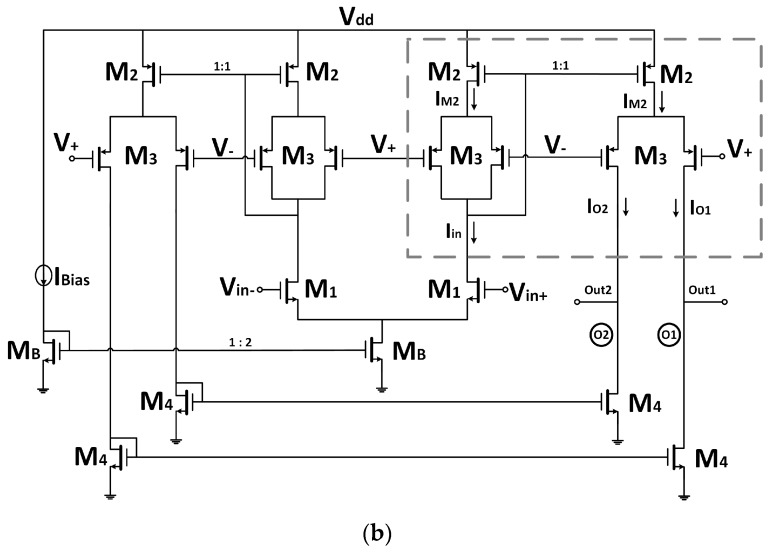
(**a**) Classic mirrored OTA; (**b**) current steering OTA.

The transconductor core was the classic mirrored Operational Transconductance Amplifier (OTA) (Figure 3a). Its overall transconductance was given by *G_m_* = k*g_m1_, with *g_m1_* the transconductance of the input differential pair M1 and k the gain factor of the current mirror. To keep an intrinsic reduced *G_m_* value, the input pair was designed to have a small *g_m1_* ~µS with a bias current I_Bias_ = 0.5 µA while unity gain (k = 1) current mirrors were used. Thus, this scheme provided the same gain *G_m_* = *g_m1_* as the classical differential pair, but uncoupled the input and output common-mode range at the cost of doubling the power consumption. Because the input voltage needs to swing from V_dd_/2 to nearly V_dd_ for our target LIA application, a Negative-channel Metal-Oxide-Semiconductor (NMOS) input pair was used.

On the basis of this structure (Figure 3a), the idea was to keep constant the input V–I conversion gain (*g_m_*) so that the input NMOS differential pair was biased with a constant bias current introduced through a 1:2 current mirror, and a current steering technique was introduced in the output current transfer section to reduce the overall *G_m_*. This was achieved by replacing the conventional M2 current mirrors by current steering gain tuneable M2–M3 high swing cascode current mirrors, as shown in Figure 3b. Transistors M2 remained equal, but cascode transistors M3—both in the input and output branches—were split into identical transistors driven not by a constant V_C_ gate voltage but by complementary control voltages V_±_ = V_C_ ± V_gc_ [39], resulting in two output branches conveying complementary currents.

Because transistors M2 present the same drain to source voltage and gate to source voltage, the current mirror operated properly, rendering unity gain current I_out_ = I_in_. The output current I_out_ was split into two complementary currents, I_O1_ and I_O2_, whose fractional value α_i_ (0 ≤ α_i_ ≤ 1) depended upon the differential control voltage V_gc_:

(4) $$\begin{array}{c} \begin{array}{c} \;I_{out}=I_{O1}+I_{O2,}\\ \;I_{O1}=\left(1-\propto_{i}\right)I_{in}, \end{array}\\ I_{O2}=\;\propto_{i}I_{in}. \end{array}\;$$

Therefore, the transconductance gain for each output had complementary values:

(5) $$\begin{array}{c} \;G_{m_{O1}}\;=\;\left(1-\propto_{i}\right)g_{m1},\;\\ \;G_{m_{O2}}\;=\;\propto_{i}g_{m1},\; \end{array}$$

with *G_m_*_o1_ + *G_m_*_o2_ = *G_m_* = *g_m_*_1_.

**Figure 4:**
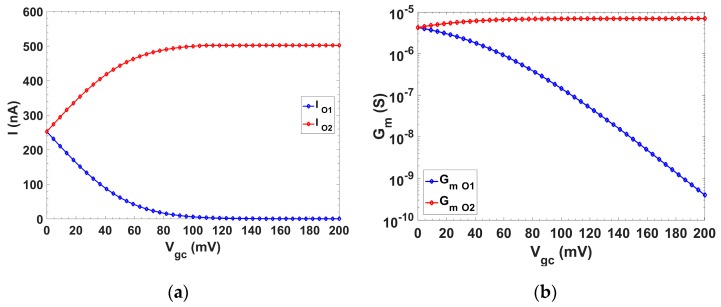
Simulated behavior of (**a**) current and (**b**) *G_m_* over V_gc_ for branches O1 and O2.

The simulated behavior of the DC current splitting over the control voltage variation is shown in Figure 4a, for V_gc_ > 0 (the figure is complementary for V_gc_ < 0). The tuning voltage variation V_gc_ ranged from 0 up to ≈200 mV, to keep the output offset bounded below ±1% and a DC gain error below 0.5 dB. Note that for V_gc_ = 0, I_O1_ = I_O2_ = I_Bias_/2 = 250 nA; for V_gc_ > 0, I_O1_ < I_O2_; for V_gc_ < 0, I_O1_ > I_O2_ and the complementary division is obtained [39].

The simulated transconductance variation in both branches O1 and O2 is shown in Figure 4b. Initially, transistors M3_O1_ and M3_O2_ were in saturation, strong inversion, and I_O1_ and I_O2_ followed a linear relation with V_gc_ (up to ≈50 mV). Transistor M3_O2_ remained in strong saturation over all V_gc_ variation, but for approximately V_gc_ > 50 mV, M3_O1_ entered the weak inversion regime, and thus its current had an exponential relationship with V_gc_ and therefore the transconductance for O1 followed a linear dependence with V_gc_ in a logarithmic scale, as can be seen in Figure 4.

**Figure 5:**
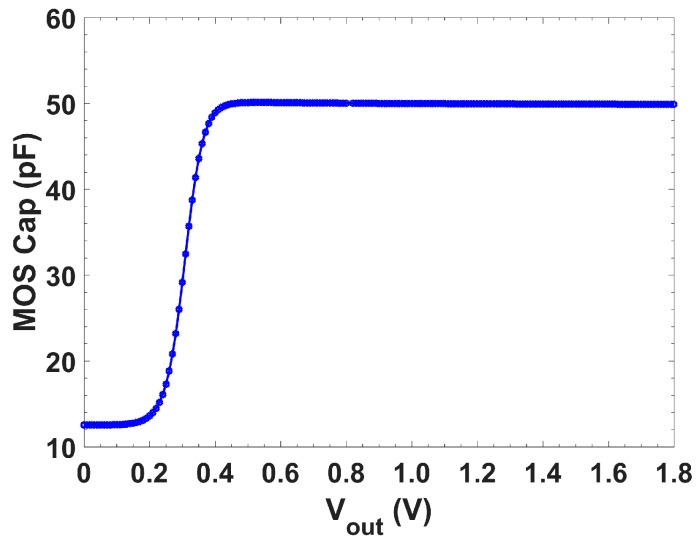
Capacitance variation of the MOS capacitor over the output voltage.

Figure 5 shows the capacitance variation of a MOS (Metal-Oxide-Semiconductor) capacitor over the output voltage, showing that from V_dd_/2 to V_dd_ it had a constant 50 pF capacitance.

### 2.2. O1-Filter: O1F

**Figure 6:**
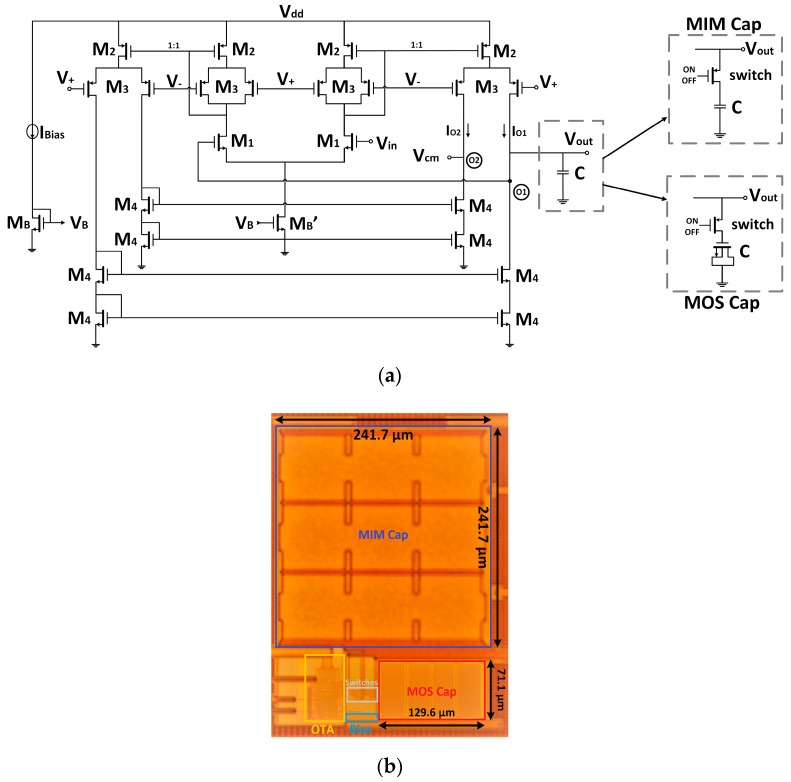
O1F (**a**) proposed integrated circuit; and (**b**) photograph. *MIM: Metal-Insulator-Metal

The schematic of the basic unity gain integrator, named O1F, is shown in Figure 6a. Transistor sizes (in µm/µm) were M1 = 7.5/10, M2 = 10/4, M3 = 5/4, M4 = 1/4, MB = 2/10, and MB’ = 4/10. It had a 1.8 V supply voltage with a common mode V_cm_ = V_dd_/2 = 0.9 V, V_C_ of the complementary control voltages at the gates was set to 1.2 V to maximize the input range, the bias current—externally generated—was set to 0.5 µA, with a total power consumption of 5.4 µW. The reason for using such lengths (L = 10 µm) was, on one hand, to reduce the input-referred noise at the differential input pair. On the other hand, in this way, a small W/L ratio can be achieved, making the input pair *g_m_* smaller (*g_m_* ≈ 10 µS) while operating in saturation with bias currents ~µA.

For the output stage, two conventional NMOS cascode current mirrors generated the complementary outputs O1 and O2.

Cascode current mirrors were chosen instead of high swing cascade, as an output voltage of 0.9 V was needed, and since the operating range for the application was from V_dd_/2 to V_dd_, an increase on the voltage range towards V_ss_ was not needed, and in this way an extra bias voltage was saved. Output O1 was selected as the integrator output, and output O2 is kept at V_dd_/2 to preserve symmetry and assure linear current division in the output branches.

Figure 6b presents the microphotograph of the integrated O1F. The active area of the proposed *G_m_* structure without the capacitor was 78.5 × 61.5 µm^2^. As shown, both a MIM (Metal-Insulator-Metal) and a MOS capacitor were implemented so that a performance comparison can be made. A clear advantage of using a MOS capacitor instead of an MIM capacitor was the great save in area (a reduction of the 85%). Thus, the total active area with the MOS capacitor was 0.0140 mm^2^.

### 2.3. O2-Filter: O2F

**Figure 7:**
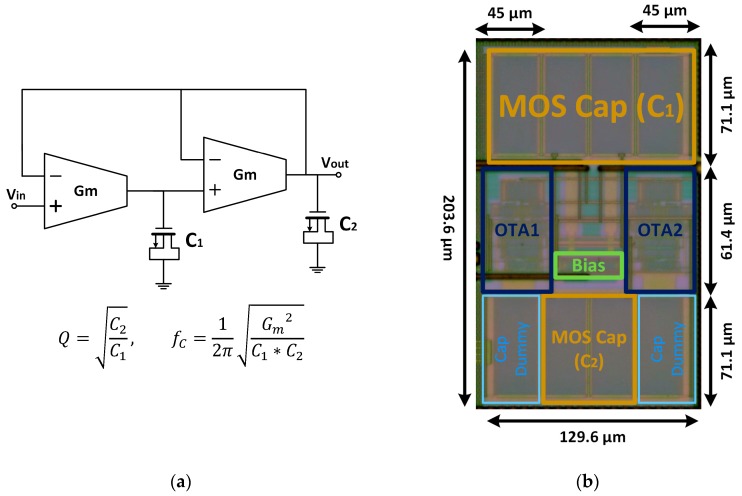
Proposed O2F (**a**) schematic with Q-factor and upper-band limit; (**b**) microphotograph.

The second order filter, named O2F, was also a unity gain scheme based on [27] with a quality factor Q = 1/√2 given by C_2_ = 0.5C_1_, C_1_ = 50 pF, both MOS capacitors. Figure 7a shows its structure as well as its quality factor and cutoff frequency, where each *G_m_* structure was identical to the one reported in the previous subsection, again with a bias current of 0.5 µA, and thus the total power consumption was 9.9 µW. The microphotograph is shown in Figure 7b.

## 3. Experimental Results

**Figure 8:**
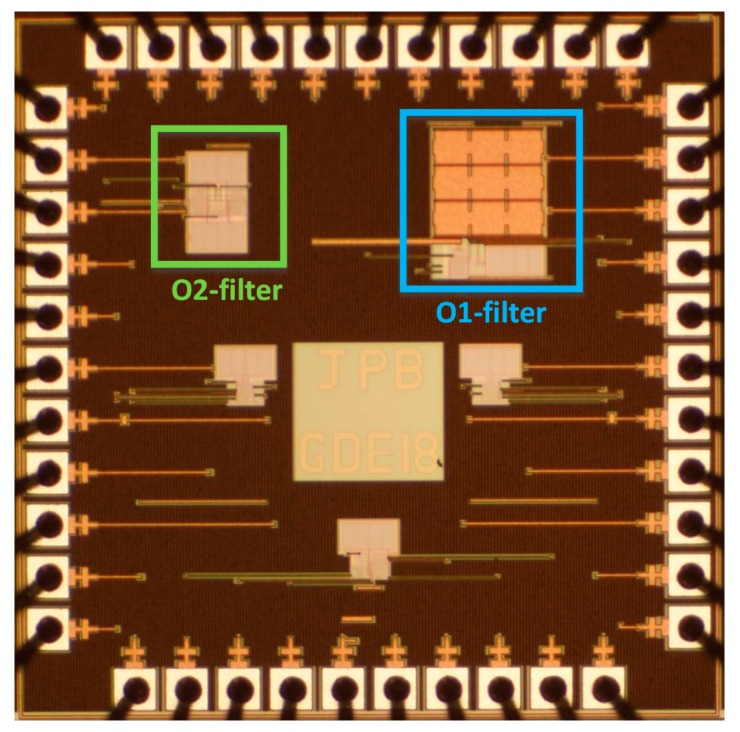
Microphotography of the integrated circuit (IC). The highlighted circuits (in blue and green) are the LPFs presented in this paper.

**Figure 9:**
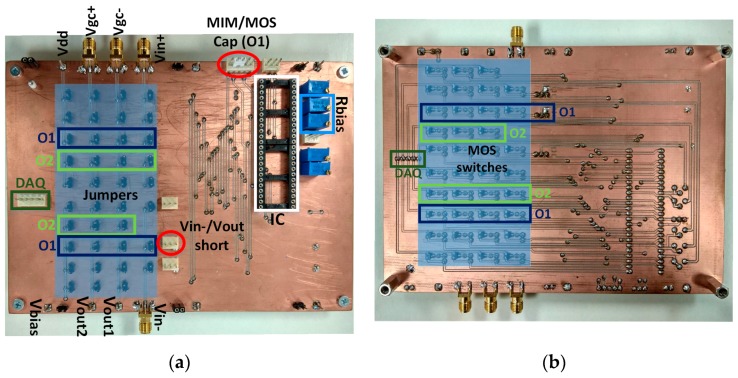
Detail of the printed circuit board (PCB) test: (**a**) front and (**b**) rear.

**Figure 10:**
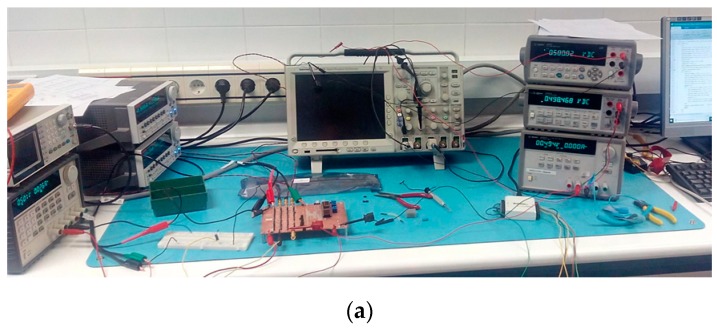

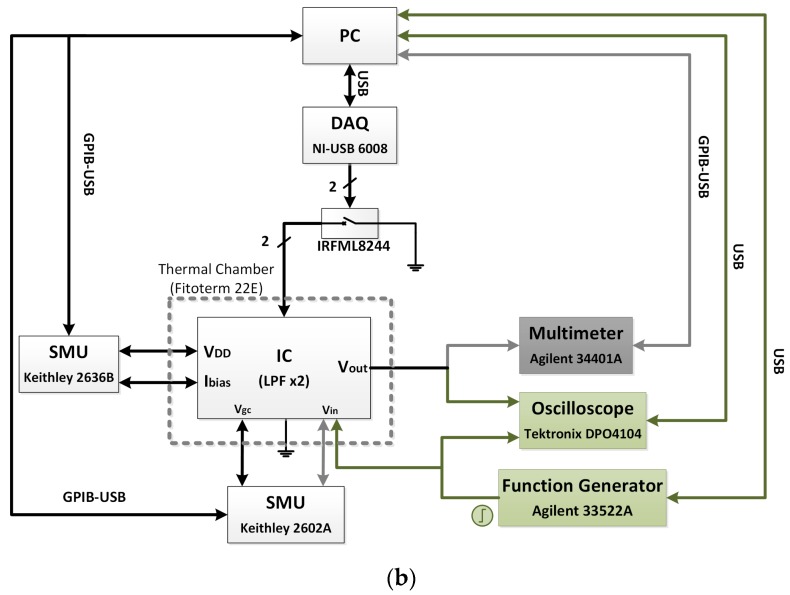
Measurement setup for the characterization of the low pass filters: (**a**) experimental setup, and (**b**) block diagram of static (grey) behavior and dynamic (green) behavior. SMU: source measurement unit, DAQ: data acquisition card.

To perform the experimental characterization of the two low pass filters, integrated in a single die (Figure 8), we designed a printed circuit board (PCB) (Figure 9). In Figure 10, the measurement setup is shown—both the experimental setup (Figure 10a) and the block diagram (Figure 10b)—for the characterization of the main parameters of the circuits: tunability, cutoff frequency range, V_in_–V_out_ characteristic, quiescent current, and linearity.

### 3.1. Experimental Setup

The integrated die had five separated circuits, as can be seen in Figure 8, with the two integrated LPFs presented in this paper being the ones marked in green and blue, using a total of 18 pins (10 for O1F, 7 for O2F and V_ss_) out of the 48 existing in the packaging used (48-DIL, Dual-In-Line). All of the circuits had a common ground, but they were biased through different input pins, as not all of them worked at the same supply voltage.

The PCB shown in Figure 9 was designed with a set of jumpers (front) and switches (rear) to select, either manually or automatically with a data acquisition card (DAQ) NI-USB 6008, the circuit to be characterized without compromising the other circuits in the die. For the switches, a low impedance NMOS transistor IRFML8244 (R_DS_ = 41 mΩ, drain-to-source resistance) with their gates connected to the digital outputs of the DAQ was used.

In addition, there were jumpers to connect the MIM or MOS capacitor and to shortcut V_in_ with V_out_ for the O1F so it could be tested as an OTA and as a filter.

One channel of a dual source measurement unit (SMU) Keithley 2636B set the voltage supply to the corresponding activated LPF, and the bias current was supplied to the circuit using the other channel. A second dual SMU was used to provide the control voltage V_gc_ and the input voltage V_in_ in the static characterization. A 34401A Agilent 6½ digital multimeter (DMM) was used to read the DC output voltage, V_out_. For the dynamic characterization, an Agilent 3352A arbitrary waveform generator (AWG) provided the input voltage, and the transient input and output signals were read through a DPO4104 Tektronix oscilloscope. All the instrumentation was connected to a PC, having the measurement process automatized. Figure 10b shows in grey the instrumentation used for the static characterization and in green the instrumentation used for the dynamic characterization.

The complementary control voltages were provided with an SMU to keep a tight control of their values and study the dependence of the filters parameters with them. However, in order to provide a portable device, this solution was not realistic, and different approaches can be employed to substitute the SMUs either using commercial components such as a digital potentiometer [40], a digital-to-analog converter (DAC) [41,42], or with a microcontroller (µC) if it is used to generate the excitation signal or to read the filtered signals from the LPFs; otherwise, it is also possible to use a specific integrated circuit (IC) to generate these voltages [43,44].

**Figure 11:**
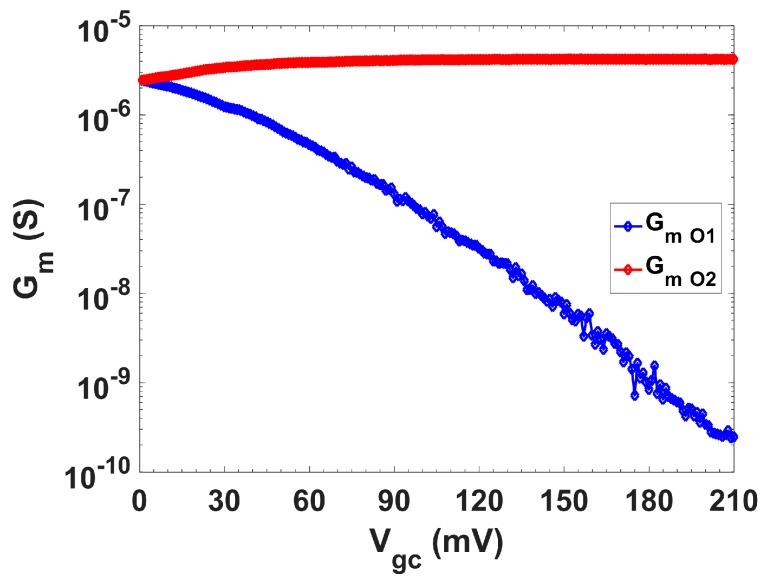
Variations over V_gc_ for *G*_m_ of branches O1 and O2 (O1F).

First, the current steering performance was validated. For this, a replica transconductor was included in the die. The current flowing through each output branch, O1 and O2, was measured by connecting a load resistor. From these measurements, the transconductances, *G_m,O1_* and *G_m,O2_*, and their dependency with V_gc_, were derived and are shown in Figure 11, presenting a good matching with the simulation results (Figure 4b).

### 3.2. G_m_-C LPF Cutoff Tunability

**Figure 12:**
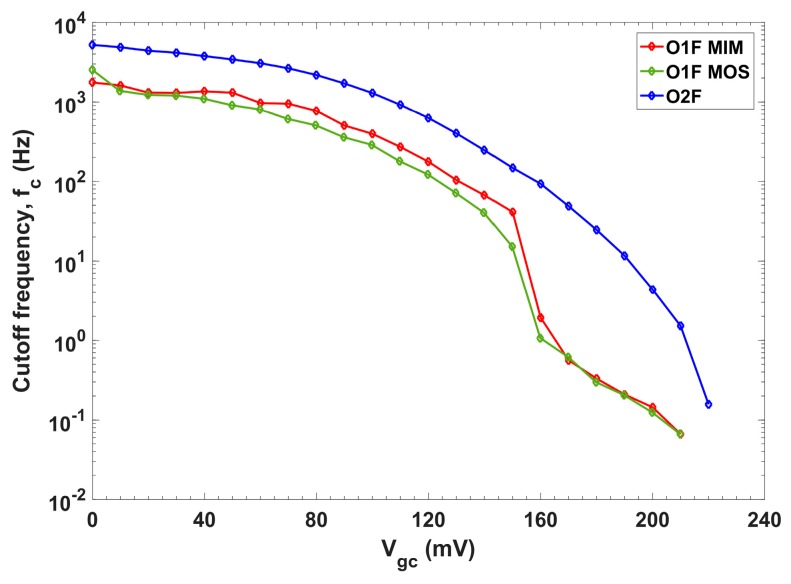
LPF cutoff frequencies for different V_gc_ values.

Figure 12 shows the filters cutoff frequencies by steeping V_gc_ in 10 mV steps. The cutoff frequency of the O1F implemented with a MOS capacitor could be tuned from 66 mHz (V_gc_ = 210 mV) up to 2.5 kHz (V_gc_ = 0 mV). On the other hand, with an MIM capacitor, the frequency range achieved was similar, from 66 mHz up to 1.2 kHz. Thus, MOS capacitor is the most suitable choice, as it rendered a comparable frequency range but with the advantage of significantly saving area. The cutoff frequency of the O2F could be tuned from 157 mHz (V_gc_ = 220 mV) up to 5.2 kHz (V_gc_ = 0 mV). Thus, the target frequencies of 0.5 and 5 Hz initially established were within the ranges of both filters.

**Figure 13:**
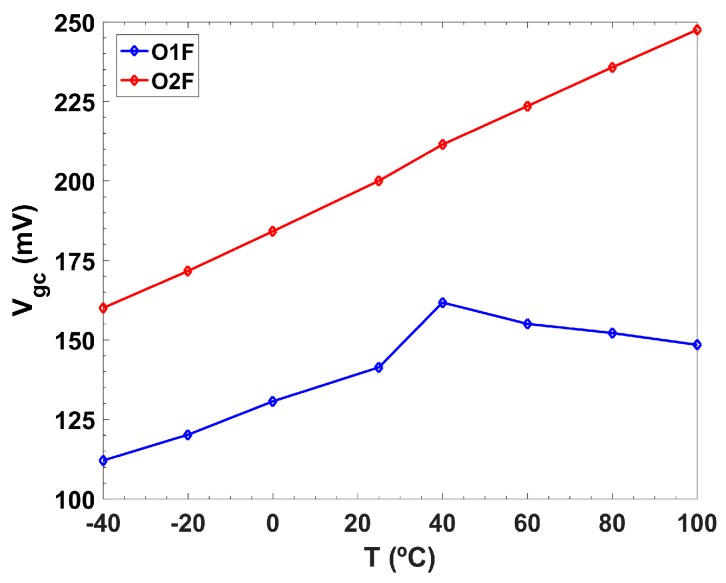
V_gc_ tuning over temperature to keep constant f_c_ at 5 Hz.

Through simulation, it was verified that both target cutoff frequencies could be met even against PT-variations (V is assumed to be provided by a voltage Low Drop-Out (LDO) regulator [45]). Experimentally, to study the influence of the temperature over the cutoff frequency, a Fitoterm 22E thermal chamber from Aralab was used to sweep the temperature from −40 to 100 °C. Despite the dependence with the temperature, it was possible to correct the variation produced by T and achieve a constant f_c_ over all of the temperature range thanks to the tunability of the circuit. In Figure 13, it was shown that the V_gc_ tuning needed to keep the cutoff frequency constant at 5 Hz for both filters.

### 3.3. DC Input/Output Characteristics

**Figure 14:**
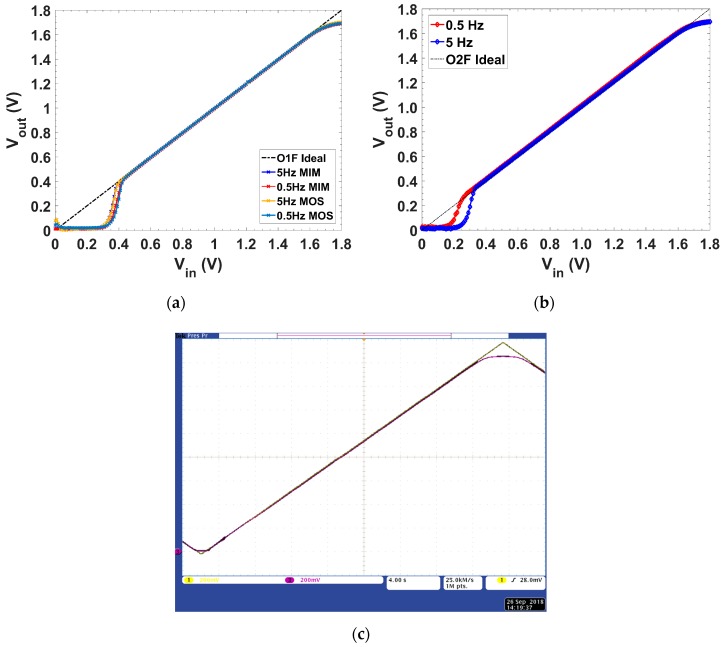
DC input/output characteristic with f_c_ 0.5 Hz and 5 Hz for: (**a**) O1F, (**b**) O2F, and (**c**) oscilloscope caption of O2F for f_c_ = 0.5 Hz. Scale (only for Figure 14c): 200 mV/square and 4 s/square.

Focusing on our two target cutoff frequencies, 0.5 Hz and 5 Hz, Figure 14 shows the static V_in_–V_out_ integrator transfer characteristic. Figure 14a,b presents detailed measurements of the input/output characteristics of filters O1F and O2F, respectively, for both target cutoff frequencies. Figure 14c presents for O2F, f_c_ = 0.5 Hz, the oscilloscope caption of the output signal for a triangular input signal ranging from 0 to V_dd_. These measurements were done following the setup for static behavior presented in Figure 10b, using the 34401A Agilent DMM to read the DC output voltage, and the 2602A Keithley SMU to generate and read the input voltage.

No major difference was appreciated by using an MIM or a MOS capacitor, but from Figure 5 it can be seen that for MOS capacitors the minimum DC output voltage to provide a 50 pF capacitance was ≈0.42 V. The linear input range was 0.43 V (0.45 V-MOS) to 1.65 V (f_c_ = 0.5 Hz), and 0.39 V (0.45 V-MOS) to 1.67 V (f_c_ = 5 Hz) for O1F (Figure 14a), whereas for O2F (Figure 14b), it ranged from 0.45 V to 1.65 V and 0.45 V to 1.67 V for f_c_ = 0.5 Hz and 5 Hz, respectively. Note that this will not affect the achieved dynamic range, as will be shown next.

### 3.4. Dynamic Range

**Figure 15:**
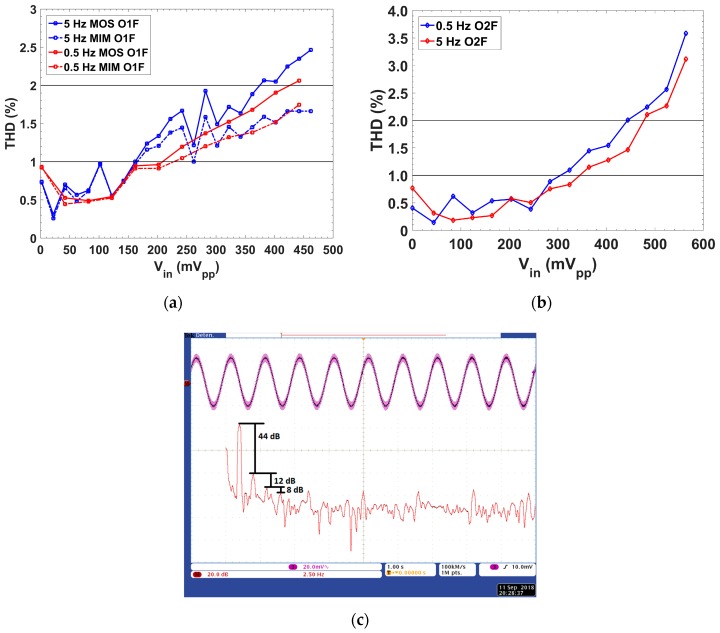
Total harmonic distortion (THD) versus input voltage peak to peak for (**a**) O1F and (**b**) O2F; and (**c**) detail of the frequency spectrum for O1F MIM-Cap. (f_c_ = 5 Hz, f_in_ = f_c_/5, amplitude 41 mV_pp_).

The total harmonic distortion (THD) as a function of the peak-to-peak amplitude is shown in Figure 15. The setup measurement followed the green setup of Figure 10b, which corresponded with the setup for the dynamic behavior. A sinusoidal input signal at a frequency f_c_/5 and with variable amplitude was generated with the 33522A Agilent AWG, whereas the DPO4104 Tektronix oscilloscope measured the output signal. The fast Fourier transform (FFT) of the output signal was recovered, computing the THD for each amplitude of the input signal.

For O1F, THD was below 1% at a frequency f_c_/5 up to 210 mV_pp_ in the cases of f_c_ = 0.5 Hz and up to 162 mV_pp_ in the cases of f_c_ = 5 Hz (Figure 15a). For O2F, THD was below 1% at f_c_/5, for amplitudes up to 305 mV_pp_ and 345 mV_pp_ for f_c_ = 0.5 Hz and 5 Hz, respectively (Figure 15b). The THD for the filter using a MIM capacitor presented similar values as with the MOS capacitor.

Figure 15c shows a detailed view of the frequency spectrum for one of the THD values shown in Figure 15a. It shows the input signal (in blue) with a 41 mV_pp_ amplitude and a 1 Hz frequency coupled with the 50 Hz line signal. The math function (in red) represents the FFT (fast Fourier transform) of the signal after being filtered by O1F with a 5 Hz cutoff frequency. From Figure 15c, after processing the FFT, the THD obtained was 0.65%, which corresponded with the O1F-5 Hz MIM-Cap. value of Figure 15a.

**Figure 16:**
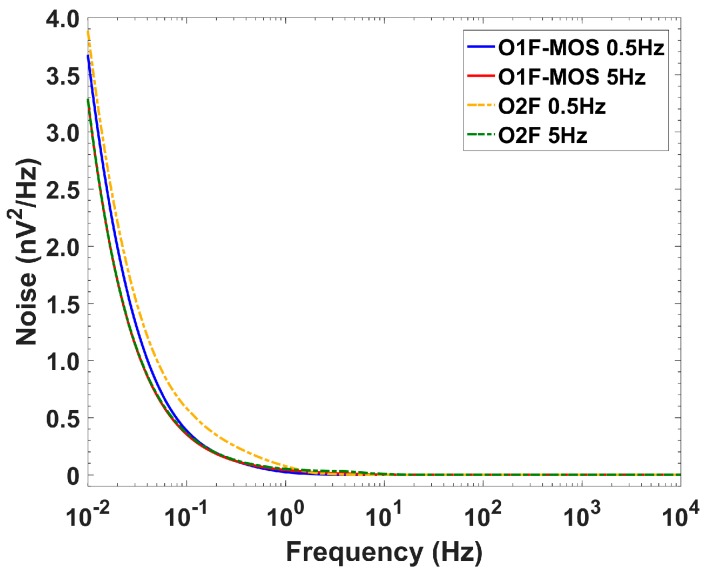
Noise over frequency for both cutoff frequencies of O1F-MOS and O2F.

The rms (root mean square) noise was obtained through simulation (Figure 16) of the extracted views of each circuit over an integration band of 10 kHz. Values for cutoff frequencies of 0.5 and 5 Hz were, respecively: 13.3 and 16.3 µV_rms_ for O1F, and 19.2 and 19.9 µV_rms_ for O2F. Thus, the dynamic range was above 70 dB for both filters and cutoff frequencies.

## 4. LPF in a Lock-In Amplifier

**Figure 17:**
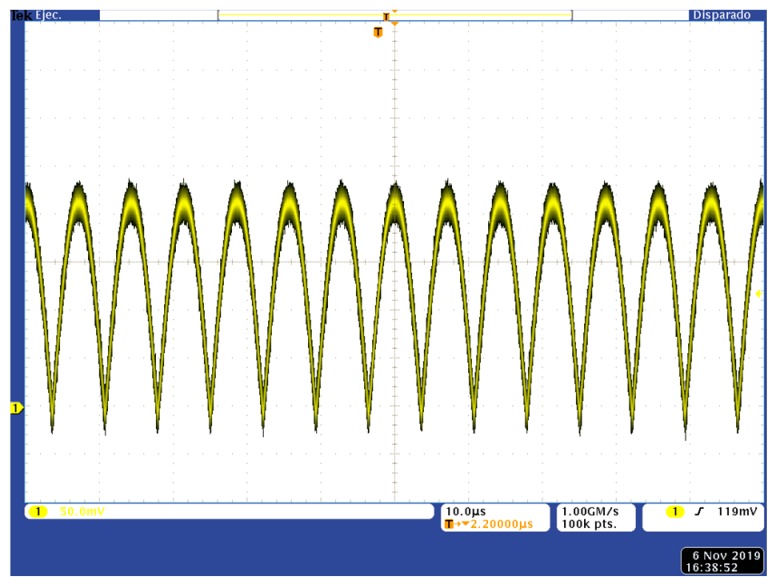
Rectified input signal for a 200 mV_pp_ amplitude with embedded white noise (signal-to-noise ratio (SNR) = 20 dB).

The proposed circuits were tested, operating as the last processing element in a lock-in amplifier—a LPF responsible for obtaining the average value of a voltage signal provided by the previous stage, a synchronous rectifier. Figure 17 shows an example of a signal provided by a synchronous rectifier prior to being filtered to recover its DC component, showing a 200 mV_pp_ noise-free amplitude embedded in white noise with a signal-to-noise ratio (SNR) of 20 dB. The frequency of the input test signals was set to 70 kHz, in the range of the resonance frequencies of the microcantilever-based sensors used in volatile organic compounds (VOC) detection and identification [46]. For the sake of simplicity, we considered test signals as being provided by purely resistive systems, where the DC value followed Equation (2), being the phase shift θ = 0, and a single-phase LIA can recover the input data, whereas for signals provided by complex impedance devices (whose phase shift θ can be nonzero), a dual-phase LIA was needed to recover both amplitude and phase information, and thus two LPFs would be required to obtain the average values given in Equations (2) and (3).

**Figure 18:**
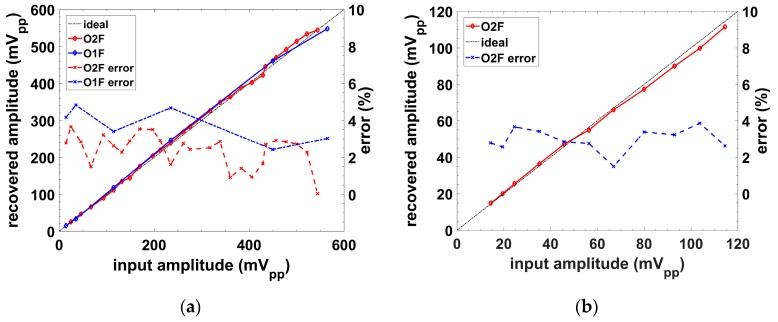
Lock-in amplifier (LIA) experimental recovered amplitude versus input signal: (**a**) amplitude values up to 560 mV_pp_ with G = 100; and (**b**) zoomed area for the first 120 mV_pp_.

Figure 18 shows the DC voltage values recovered for input signals with amplitude values (peak-to-peak) ranging from 150 µV to 5.75 mV, and an SNR > 20 dB. Signal was previously conditioned by a preamplifier with a gain G = 100. The LPF cutoff frequency was set to f_c_ = 5 Hz. Selecting a cutoff frequency 10 times lower (f_c_ = 0.5 Hz), the recovered amplitude would present a higher accuracy but at a much longer output stabilization time. Similarly, a higher f_c_ would provide a faster response but at a lower accuracy.

The proposed O1F filter presented a current consumption below 3 µA, a tuneable cutoff frequency spanning over five orders of magnitude, and an area of 0.0140 mm^2^; otherwise, the O2F filter provided a better average voltage estimation but at an increase of power and area consumption. Table 1 shows a comparison with previously reported works covering similar tunability and frequency ranges to our proposals. Analyzing the figures of merit (FoMs) in the literature, we found that the main parameters that are involved are power, dynamic range (DR), order of the filter (n), bandwidth (BW), and area consumption. We included in the table two FoMs defined in [31,32], as they not only take into account all the previous parameters, but also normalize the power (NP) and the area (NA) consumption to the technology used, according to:

(6) $$FoM_{1}=\frac{NP}{n*DR}\;$$

(7) $$FoM_{2}=\frac{Power*BW*NA}{n*DR}$$

with NP = Power × [0.5/(V_dd_ − V_th_)] × (1/V_dd_) and NA = area(mm^2^)/Tech(µm^2^)^2^, with V_th_ = 0.4 V for 0.18 µm CMOS technology and 0.6 V for 0.35 µm CMOS technology.

As Table 1 shows, both filters presented a significant enhancement in the dynamic range, whereas the target cutoff frequencies can be maintained for a range of temperatures from −40 °C to 100 °C. Both FoMs showed a good performance for all the frequency range compared with the other proposed filters, proving it was an efficient solution in terms of power and area consumption.

## 5. Conclusions

Two low pass filters with programmable cutoff frequencies were presented in this paper. They relied on a simple current steering technique to give tunability over a wide frequency range. The first order LPF with a 1.8 V voltage supply presented a five orders of magnitude f_c_ range, with a low power consumption and a high dynamic range. Similar results were achieved with the second proposed structure, a second order design, with a 1.8 V voltage supply, increasing the cutoff frequency range and slightly enhancing the dynamic range at the expense of an increase of the area and power consumption. Compared to state-of-the-art solutions, the proposed structure exhibited very competitive performances while meeting the critical requirements of battery-portable on-chip micro-instruments in terms of power and area efficiency, critical for its implementation in multichannel measurement instruments for impedance sensor arrays. Further reduction on the power consumption could be achieved by lowering the bias current used.

The recovered signals from Section 4 showed the validity of the proposed LPFs as DC magnitude extractors. Higher accuracy over the recovered signal was achieved with the second order filter, although a higher consumption in power and area was required. Thus, there was an accuracy power–area trade-off that was dependent on the order of the filter. Thanks to the implementation of a fully integrated low pass filter, we achieved a completely integrated lock-in amplifier, together with the previously authors proposal [21] for a multichannel measurement device.

## Figures and Tables

**Table 1:** LPF performance comparison with similar *G_m_*-C works.

Parameter	O1F	O2F	[24] ‘14	[25] ‘15	[47] ‘15	[33] ‘18	[32] ‘18	[23] ‘18
Technology (µm)	0.18	0.18	0.6	0.35	0.13	0.35	0.18	0.35
V_supply_ (V)	1.8	1.8	3.3	3.3	1.2	0.6	1	1.8
I_Bias_ (nA)	500	500	NA	NA	NA	1.5–4.5	NA	14.9–182.3
Power (µW)	5.4	9.9	75.9	59.5–90	450	9–27(10^−4^)	0.35	0.1–1.31
Order	1	2	2	9	3	4	5	2
Gain (dB)	<0.5	<0.5	≈0	18.8/21.1@f_c_	10	−2.77	−6/−8	0–12
Area (mm^2^)	0.0140	0.0264	0.17	0.9	0.08	0.168	0.12	0.12
T range (°C)	−40 to 100	−40 to 100	NA	NA	NA	NA	NA	NA
DC in/out range (V)	0.39(0.45 **)–1.65	0.45–1.65	NA	NA	NA	NA	NA	NA
f_c_ (Hz)	0.066–2.5 k	0.157–5.2 k	2.5 k–10 k	31–8 k	375 k–590 k	101–272	50	2 k–20 k
noise (µV_rms_)	13.3; 16.3^(a,b)^	19.2; 19.9^(a,b)^	91.8; 60.7	93.3; 34.3^(c)^	342	46.6; 46.8	100	86.3; 84.3
V_pp_@THD≤1%	0.22; 0.16^(a)^	0.305; 0.345^(a)^	4.13; 3.13	0.082; 0.031^(d)^	0.45	NA	NA	0.216; 0.294
DR (dB)	75.3; 70.9^(a)^	75; 75.7^(a)^	84–85.2	49.8–50.2	53.35	47	49.9	58.9; 61.8
NP (µ)	1.07	1.96	NA	3.34–5.05	NA	NA	0.292	0.02–0.3
Normalized Area	0.432	0.815	0.472	7.347	4.734	1.371	3.704	0.980
FoM_1_ (10^−10^)	1.838–3.051	1.743–1.608	NA	12–17.34	NA	NA	1.868	0.114–1.219
FoM_2_ (µ)	2.64*10^−5^–1.66	1.126 × 10^−4^–3.44	2.83–9.84	4.87–1.816 × 10^3^	0.573 × 10^6^–0.9 × 10^6^	1.39 × 10^−4^–1.12 × 10^−3^	0.0415	0.11–10.4

* NA: not available, DR: dynamic range, NP: normalization of power, FoM: figures of merit; ** minimum linear range with MOS capacitor; **^(a)^** for f_c_ = 0.5 and 5 Hz, respectively; **^(b)^** simulated; **^(c)^** minimum noise values; **^(d)^** @THD < 5%.
